# The Anti-Glioma Effect of Juglone Derivatives through ROS Generation

**DOI:** 10.3389/fphar.2022.911760

**Published:** 2022-06-14

**Authors:** Jinsen Zhang, Minjie Fu, Jinfeng Wu, Fengfeng Fan, Xin Zhang, Chunjie Li, Hui Yang, Yonghe Wu, Yiming Yin, Wei Hua

**Affiliations:** ^1^ Department of Neurosurgery, Huashan Hospital, Fudan University, Shanghai, China; ^2^ National Center for Neurological Disorders, Shanghai, China; ^3^ Shanghai Key Laboratory of Brain Function Restoration and Neural Regeneration, Shanghai, China; ^4^ Neurosurgical Institute of Fudan University, Shanghai, China; ^5^ Shanghai Clinical Medical Center of Neurosurgery, Shanghai, China; ^6^ Department of Dermatology, Huashan Hospital, Fudan University, Shanghai, China; ^7^ Shanghai Institute for Advanced Immunochemical Studies, ShanghaiTech University, Shanghai, China; ^8^ Departmeng of Neurosurgery, The Affiliated Suzhou Hospital of Nanjing Medical University, Suzhou, China

**Keywords:** glioblastoma, juglone, chemotherapy, ROS, apoptosis

## Abstract

Juglone has been extensively reported as a natural antitumor pigment. However, it is easy to be oxidized due to active hydroxy in the quinone. Here, we designed some new juglone derivatives, as the hydroxy was replaced by methyl (D1), allyl (D2), butyl (D3), and benzyl (D4) groups. Nuclear magnetic resonance spectra and mass spectrometry were applied to confirm the derivatives and oxidative products of juglone. U87 and U251 cell lines were used for tests *in vitro*, and primary human glioblastoma cells were applied for *in vivo* experiments. The CCK8 and EdU assay demonstrated the anti-tumor effect of the four derivatives, and IC50 for U87 was 3.99, 3.28, 7.60, and 11.84 μM, respectively. In U251, IC50 was 7.00, 5.43, 8.64, and 18.05 μM, respectively. D2 and D3 were further selected, and flow cytometry showed that apoptosis rates were increased after D2 or D3 treatment via ROS generation. Potential targets were predicted by network pharmacology analysis, most of which were associated with apoptosis, cell cycle, and metabolism pathway. CDC25B and DUSP1 were two of the most likely candidates for targets. The orthotopic glioblastoma model was established to evaluate the anti-glioma effect and side-effect of juglone derivatives, and the *in vivo* experiments confirmed the anti-glioma effects of juglone derivatives. In conclusion, new derivatives of juglone were created via chemical group substitution and could inhibit glioma cell viability and proliferation and induce apoptosis rate *via* ROS generation.

## Introduction

Glioma is the most common primary malignant brain tumor, and glioblastoma (GBM) contributes 50–60% of them. Despite the advance in molecular research of GBM, the overall survival remains as poor as 14.6 months even after comprehensive management (Stupp et al., 2005; Zhu et al., 2017). Temozolomide (TMZ) was demonstrated as a first-line chemotherapeutic agent through DNA alkylation by clinical trials, but GBM would resist TMZ when MGMT is unmethylated or when the tumor recurs (Newlands et al., 1997; Yung et al., 2000; Stupp et al., 2001). Tumor treating fields (TTFields) also could partially benefit GBM patients (Zhu et al., 2017). However, many endeavors such as anti-VEGFA (Batchelor et al., 2014), anti-EGFRvIII (Schuster et al., 2015), and anti-PDL1 (Berghoff et al., 2015) all failed to meet the set goals. Therefore, there is still urgency to develop new therapeutic approaches for GBM.

Juglone shows broad anti-cancer activity in traditional herbal medicine (Sugie et al., 1998; Xu et al., 2012; Redaelli et al., 2015; Zhang et al., 2015). It has been reported that juglone could exert its anti-glioma effect for its fat-soluble characteristics *in vitro* and *in vivo* in human GBM cells (Wu et al., 2017) and also in C6 rat glioma cells (Meskelevicius et al., 2016). It is also cytotoxic to human leukemia, cervical carcinoma, and pancreatic cancer cells (Xu et al., 2012; Zhang et al., 2012; Avci et al., 2016). The potential mechanism includes the activation of the apoptotic caspase cascade and the accumulation of intracellular reactive oxygen species (ROS) (Sajadimajd et al., 2016). Juglone is also taken as a PIN-1 (peptidyl-prolyl cis/trans isomerase 1) inhibitor for malignant solid tumors (Xu et al., 2016).

Although the anti-glioma effect was confirmed in our previous report (Wu et al., 2017), there are still some concerning issues. The preservation of juglone is difficult due to its instability and susceptibility to oxidation, which could decrease the antitumor effects. Hence, new derivatives of juglone are designed to increase stability and lipophilicity, with the hydroxy-substituted by other chemical scaffolds, such as methyl, allyl, butyl, and benzyl group. The toxicity and potential mechanism of antitumor effects are also explored in this study both *in vitro* and *in vivo*.

## Methods and Materials

### Chemical Synthesis of New Juglone Derivatives

New juglone derivatives (D1-D4) were prepared according to literature procedures (Clive et al., 2004; Mitchell et al., 2013; Li and Shen, 2020). Ag_2_O (117 mg, 0.5 mmol) and alkyl halide (1.5 mmol) was added to the solution of juglone (174 mg, 1 mmol) in CH_2_Cl_2_ (5 mL). The reaction mixture was stirred at room temperature for 24 h. After filtration through celite and removal of the solvent in vacuo, the residue was subjected to flash column chromatography on silica gel (230–400 mesh) using *n*-hexane/ethyl acetate as eluent to give the product D1-D4.

### Identification of Derivatives With Nuclear Magnetic Resonance Spectra and Oxidative Products With Mass Spectrometry

All reactions were carried out in oven-dried glassware under an atmosphere of dry N_2_ with the rigid exclusion of air and moisture using standard Schlenk techniques. Dichloromethane was freshly distilled from CaH_2_ immediately before use. All other chemicals were purchased from either J&K Chemical Co. or used as received unless otherwise specified. ^1^H and ^13^C{^1^H} NMR (Nuclear magnetic resonance) spectra were recorded on a Varian Inova 400 spectrometer at 400 and 100 MHz, respectively. All signals were reported in ppm unit with references to the residual solvent resonances of the deuterated solvents for proton and carbon chemical shifts. Mass spectra were obtained on a Thermo Finnigan MAT 95 XL spectrometer, Shanghai Institute of Organic Chemistry, CAS.

### Cells and Culture

GBM primary cells were isolated by specimens derived from patients in Huashan Hospital with full consent after approval from the local ethic committee. The molecular pathology of the specimen for *in vivo* experiment is IDH-wildtype, MGMT unmethylation, and TP53 mutation. U251 and U87 were purchased from China Academia Sinica Cell Repository (Shanghai, China). Primary GBM cells and glioma cell lines were cultured in Dulbecco’s modified Eagle’s Medium (DMEM; HyClone, Logan, UT, United States) supplemented with 10% fetal bovine serum (FBS; Gibco BRL, Gaithersburg, MD, United States). Cell cultures were maintained in a 5% CO_2_ humidified incubator at 37°C.

### Isolation and Culture of GBM Cells

GBM specimen was placed in ice and transferred to the lab within 1 h after surgical resection. The specimen was washed with PBS to remove the blood and necrotic tissue. Then the minced GBM tissue by a surgical knife blade was digested with 0.25% trypsin in the falcon tube at 37°C for 15 min and shaken every 5 min. The tissues were triturated into single cells by a 5 ml pipette and a 40 μm filter was used to remove tissue debris. Single cells were centrifuged and resuspended with 1 ml RBC lysis buffer at room temperature for 5 min. At last, GBM cells were resuspended and cultured in DMEM with 10% FBS. For subculture, cells were passaged once they reached 80–90% confluence. An intracranial implantation experiment was performed on cells within passages two and five to minimize genetic mutation.

### Cell Viability and Proliferation Assays

Juglone (Sigma, America) and derivatives were dissolved in dimethyl sulfoxide (DMSO) and diluted in DMEM. Cell viability was assessed by the Cell Counting Kit-8 assay (CCK-8, Dojindo, Japan). Briefly, tumor cells that were cultured in DMEM with 10% FBS were seeded in 96-well plates at a density of 1×10^4^ cells/100ul/well and incubated overnight. Tumor cells were pretreated with and without NAC (2 mM, Beyotime, China) for 1 h. After treatment of juglone or its derivatives in different concentrations for 48 h, cells were incubated for 1 h with 10 μl CCK-8 per well. The optical density (OD) was measured at 450 nm with a microplate spectrophotometer (Bio-Rad, United States). Proliferation was examined using the EdU incorporation (Ribobio, China) assay, which was performed according to the manufacturer’s protocol, and the cells were examined under a fluorescence microscope. The experiment was triplicated, and each contained six replicates.

### Flow Cytometric Analysis of Apoptosis

For apoptosis assay, glioma cells were treated with two kinds of juglone derivatives for 48 h, 1×10^5^ cells were harvested, resuspended in 100 µl binding buffer, and incubated with 5 µl Annexin V-FITC, 10 µl of PI (BD, San Joe CA) in darkness at room temperature for 15 min. After that, 400 µl of binding buffer was added before being tested on a FACS Calibur cytometer (BD, San Joe CA). FlowJo software (Tree Star, Ashland OR) was used to analyze the data. The experiments were triplicated.

### Measurement of Reactive Oxygen Species (ROS) Generation

DCFDA fluorescent assay (Sigma Aldrich, United States) was used to label intracellular reactive oxygen species (ROS) and then detected with flow cytometry. Briefly, 5×10^5^ cells were pretreated with different concentrations of juglone derivatives for 6 h and then loaded with DCFDA (10 μM) probe. After incubation at 37°C for 30 min, cells were harvested, washed, resuspended with PBS, and fluorescence intensity was measured by flow cytometry. FlowJo software was used to analyze the mean fluorescence intensity (MFI). The experiments were repeated three times.

### Network Pharmacology Construction and Target Prediction

The potential targets of juglone (SMILES: C1 = CC2 = C(C(=O)C=CC2 = O)C (=C1)O) were obtained from SwissTargetPrediction (http://swisstargetprediction.ch/). The top 100 genes were selected for the construction of a protein-protein interaction (PPI) network (https://string-db.org/). The TSV format file was downloaded from string and imported into Cytoscape software (version 3.8.0) for visualization. Molecular docking was performed using UCSF Chimera (Pettersen et al., 2004). The structure data of CDC25B and DUSP1 for docking were obtained from alpha fold (https://alphafold.com/) (Jumper et al., 2021; Varadi et al., 2022).

### Western Blot Assay

After treating with different concentrations of juglone derivatives for 48 h, total protein of glioma cell lines of U87 and U251 were obtained from RIPA lysis buffer with 1% PMSF (Beyotime, China). The protein concentration was determined by BCA assay (Beyotime, China), and samples were separated on 10% SDS-PAGE, and then transferred onto NC membranes (0.45 μm, Millipore, United States). The membranes were incubated with primary Abs against cleaved-PARP (1:1000, Cell Signaling Technology, China) and β-actin (1:10000, Cell Signaling Technology, China) overnight at 4°C, followed by HRP conjugated secondary Ab (1:3000, Cell Signaling Technology, China). The protein bands were visualized using enhanced chemiluminescence (ECL, Millipore, United States) and a detection system (ChemiDoc Touch, Bio-Rad)

### Cytotoxicity of Juglone Derivatives on Glioma Cells *In Vivo*

All animal procedures were conducted according to protocols approved by the Institutional Animal Care and Use Committee at Fudan University.

Female BALB/c-nu mice (3–4 weeks old) provided by SLAC Laboratory Animal Company (Shanghai, China) were used as orthotopic xenograft recipients. Mice were housed in an environment with a 12-h light/dark cycle. At least 1 week time was provided for mice to acclimatize new environment before experimentation. Human primary GBM cells were labeled with lentivirus expressing luciferase. Before intracranial transplantation, cells were digested into single cells and suspended with PBS at a density of 1×10^5^ cells/μL. Mice were anesthetized intraperitoneally with 10% chloral hydrate and secured into a stereotaxic apparatus. GBM cells in 10 uL PBS *via* a Hamilton syringe were injected into the right forebrain (2.5 mm lateral and 1 mm anterior to bregma, at a 2.5 mm depth from the skull surface). The mice were randomly divided into three groups (control group, D2, and D3 treatment group). The number in each group was five. D2 and D3 were dissolved in DMSO and diluted in PBS; the final concentration of DMSO was 20 mg/ml. PBS containing the same concentration of DMSO was used as vehicle control. Juglone derivatives treatment group was injected intraperitoneally with D2 and D3 (1 mg/kg) every 2 days, which was the same as the previous dosage. Bioluminescent imaging was performed on the twenty-eighth day after transplantation with IVIS-200 (Xenogen, United States) to test the tumor volume. Before anesthesia, D-luciferin (Yeason, China) was injected intraperitoneally at 150 mg/kg body weight. Images of different groups were captured with the same parameters. Bioluminescence values of intracranial tumors were quantitated using the Living Image software. Mice were euthanized when neurological symptoms appeared and perfused with 4% paraformaldehyde in PBS.

### Hematoxylin and Eosin Staining

The whole brain, heart, liver, and kidney of each mouse were collected. Fixation with 4% paraformaldehyde in PBS, dehydration with gradient ethanol, embedded in paraffin, and section cut in 5 μm thickness were performed. The sections were stained with hematoxylin and eosin (H&E). Cell morphology of different tissue was observed under a light microscope.

### Statistical Analysis

All quantified data were presented as mean ± SEM. For comparison between the two groups, two-tailed student’s t-tests were used to calculate *p* values. A *p* value < 0.05 was considered statistically significant.

## Results

### Juglone Was Gradually Oxidized in a Time-Dependent Manner

**FIGURE 1:**
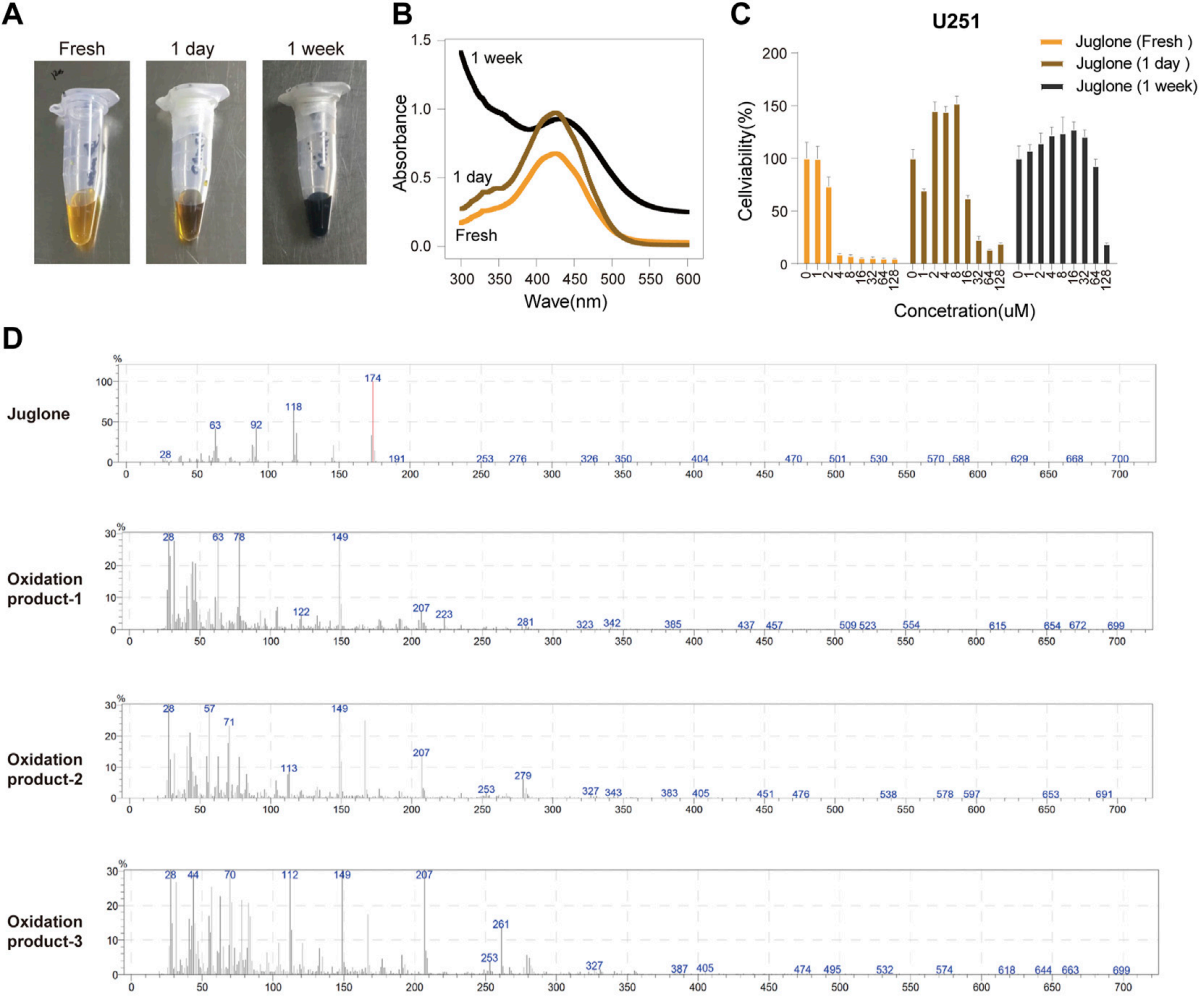
Juglone was oxidized in a time-dependent manner. **(A)** A dark brown color change of juglone occurred in a time-dependent manner. **(B)** The maximum absorbance wavelength widened as juglone was oxidized. **(C)** The cytotoxic effects of juglone decreased dramatically after being oxidation. **(D)** Mass spectrometry revealed several unknown oxidation products of juglone.

The phenomenon that juglone is easy to be oxidated has previously been observed. Juglone solution took on dark brown color changes in a time-dependent manner when preserved at 4°C in an EP tube (Figure 1A). The maximum absorbance wavelength of the samples changed, which indicated several compounds existed after oxidation (Figure 1B). The cytotoxicity of juglone oxidation products was assessed by using the CCK-8 assay. Interestingly, juglone oxidation products had pro-tumor effects in low concentrations and antitumor effects in high concentrations. The cytotoxic effects of juglone were decreased dramatically in the same concentration after oxidation (Figure 1C). To figure out what the oxidation product was, we tested the solutions of juglone in fresh, 1 day, and 1 week after dissolution by using mass spectrometry (Supplementary Material S1). The peak of juglone could be detected in fresh solution (Molecular Weight: 174kD), while many unknown compounds could be found, and juglone itself decreased obviously after oxidation (Figure 1D).

### Juglone Derivatives Were Synthesized in the Chemical Method

**FIGURE 2:**
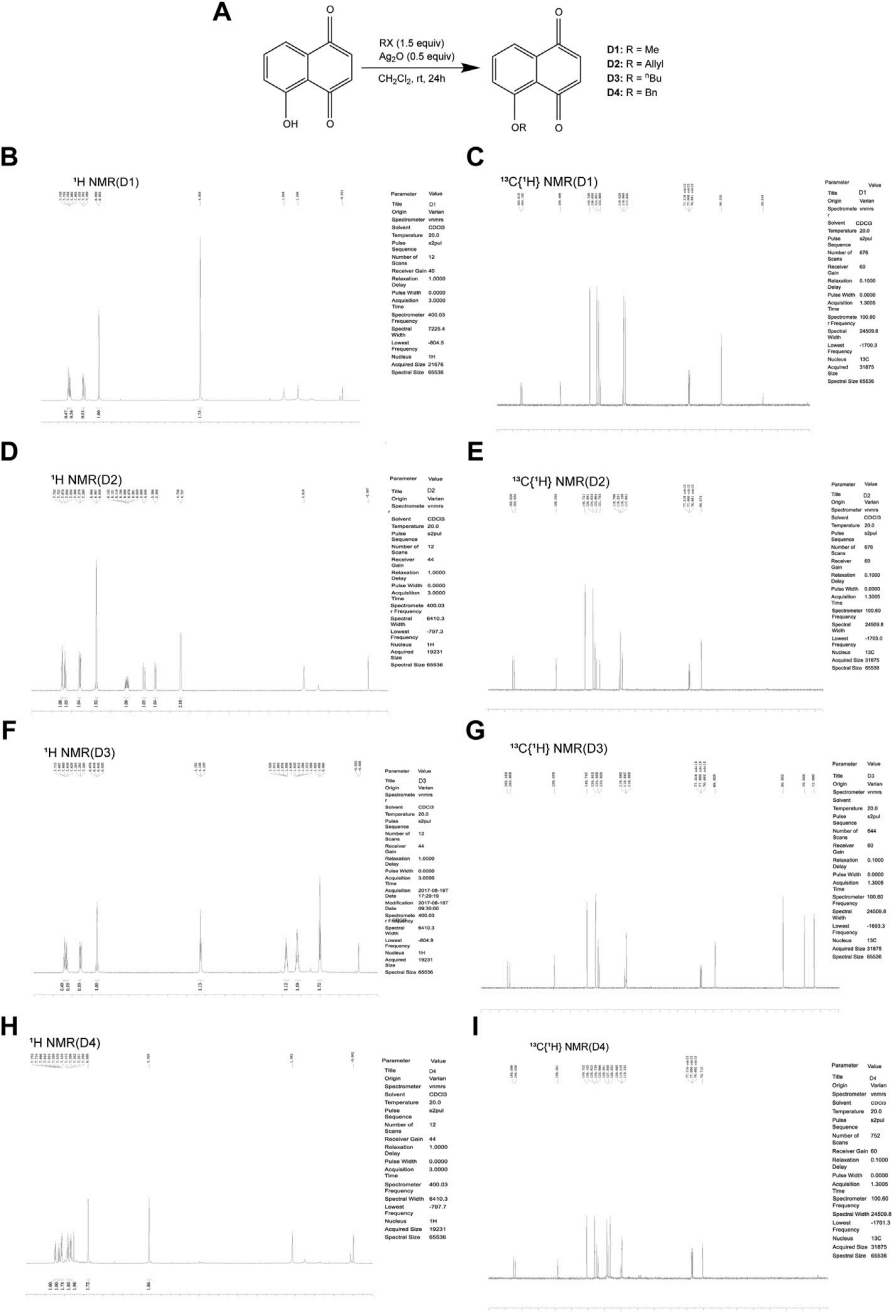
Chemical synthesis of juglone derivatives and identification with NMR spectra. **(A)** Chemical synthesis process of juglone derivatives. **(B)(D)(F)(H)** Identification of juglone derivatives with 1H NMR spectra (D1, D2, D3, D4 respectively). **(C)(E)(G)(I)** Identification of juglone derivatives with ^13^C{1H} NMR spectra **(D1, D2, D3, D4 respectively)**.

The chemical protocol of synthesizing the target compounds is shown in Figure 2A. Compounds D1-D4 showed the characteristic peaks in 1H NMR spectra at corresponsive ppm respectively. 5-Methoxy-1,4-naphthoquinone (D1): Yellow solid. Yield: 84%. ^1^H NMR (400 MHz, CDCl_3_): *δ* 7.70 (m, 2H), 7.31 (d, *J* = 8.4 Hz, 1H), 6.86 (m, 2H), 4.00 (s, 3H). These data are identical with those reported in the literature (Mitchell et al., 2013) (Figure 2B, Figure 2C). 5-Allyloxy-1,4-naphthoquinone (D2): Yellow solid. Yield: 82%. ^1^H NMR (400 MHz, CDCl_3_): *δ* 7.73 (d, *J* = 7.6 Hz, 1H), 7.65 (t, *J* = 8.4 Hz, 1H), 7.29 (d, *J* = 8.4 Hz, 1H), 6.87 (m, 2H), 6.10 (m, 1H), 5.66 (d, *J* = 17.6 Hz, 1H), 5.37 (d, *J* = 10.4 Hz, 1H), 4.73 (m, 2H). These data are identical with those reported in the literature (Clive et al., 2004) (Figure 2D, Figure 2E). 5-Buthoxy-1,4-naphthoquinone (D3): Brown oil. Yield: 81%. ^1^H NMR (400 MHz, CDCl_3_): *δ* 7.70 (d, *J* = 7.2 Hz, 1H), 7.65 (t, *J* = 8.0 Hz, 1H), 7.29 (d, *J* = 8.4 Hz, 1H), 6.85 (m, 2H), 4.14 (t, *J* = 6.4 Hz, 2H), 1.89 (m, 2H), 1.60 (m, 2H), 1.01 (t, *J* = 7.6 Hz, 3H). ^13^C{^1^H} NMR (100 MHz, CDCl_3_): *δ* 185.2, 184.0, 159.1, 140.7, 135.9, 134.7, 133.8, 119.6, 118.8, 118.7, 69.0, 31.0, 19.0, 13.7. HRMS (EI) Calcd for C_14_H_15_O_3_^+^ (M + H^+^): 231.1016. Found: 231.1019 (Figure 2F, Figure 2G). 5-Benzyloxy-1,4-naphthoquinone (D4): Orange solid. Yield: 86%. ^1^H NMR (400 MHz, CDCl_3_): *δ* 7.74 (d, *J* = 7.6 Hz, 1H), 7.65 (t, *J* = 8.4 Hz, 1H), 7.58 (d, *J* = 7.6 Hz, 2H), 7.42 (t, *J* = 8.4 Hz, 2H), 7.34 (d, *J* = 8.4 Hz, 2H), 6.89 (s, 2H), 5.30 (s, 2H). These data are identical with those reported (Li and Shen, 2020) (Figure 2H, Figure 2I). The purity of compounds (>95%) was measured with GC-MS (Supplementary Material S2).

### New Derivatives of Juglone Could Exert a Cytotoxic Effect Against Gliomas *In Vitro*

**FIGURE 3:**
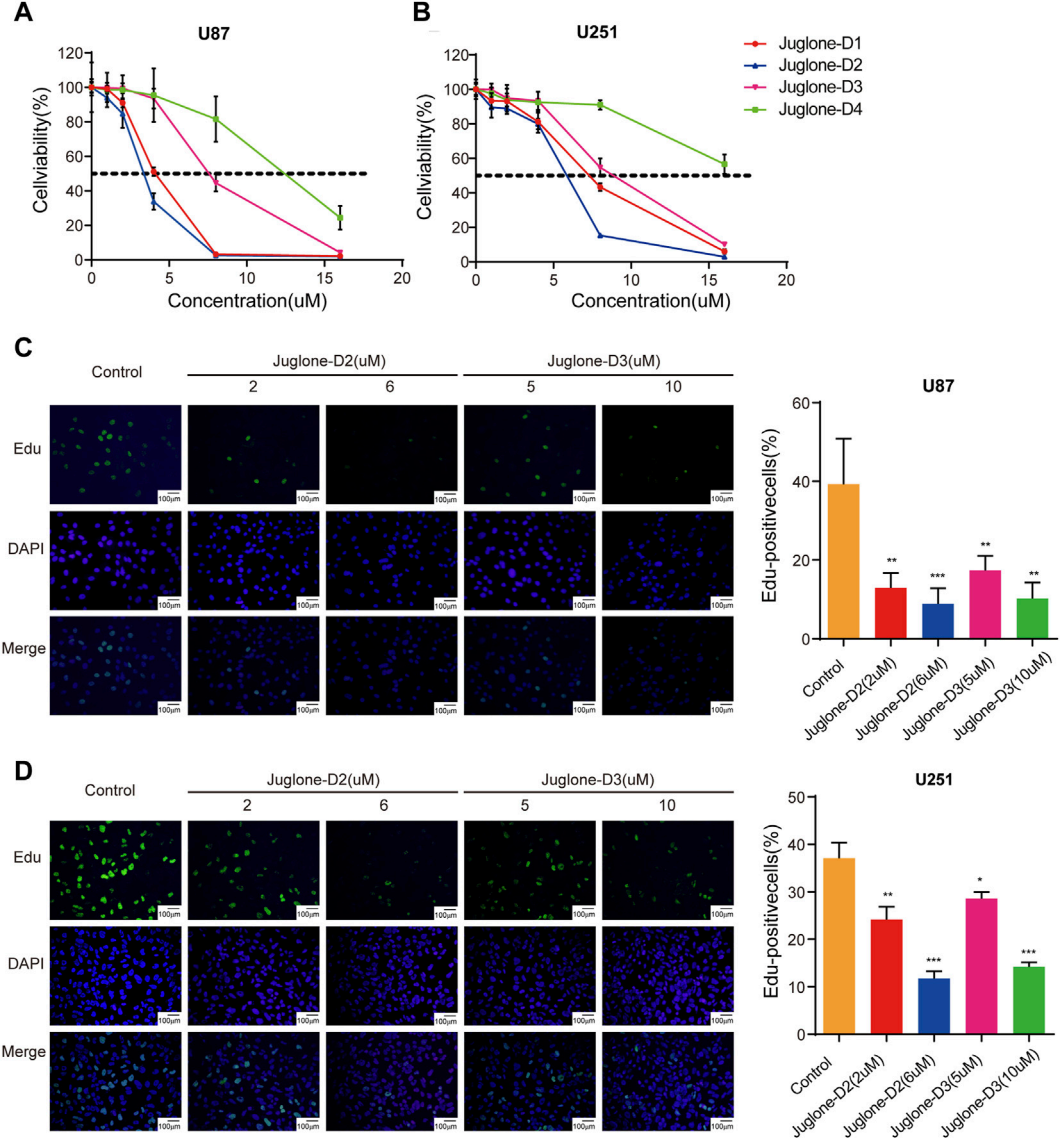
Juglone derivatives could exert a cytotoxic effect against glioma cells. **(A-B)** CCK-8 assay showed D1, D2, and D3 could inhibit cell viability of U87 and U251 cell lines. **(C-D)** Edu assay revealed D2 and D3 could inhibit the proliferation of U87 and U251 cell lines. *p<0.05, **p<0.01, ***p<0.001.

Cell viability of U87 and U251 were evaluated by CCK-8 assay after treatment with four juglone derivatives for 48 h. As shown in Figure 3A and Figure 3B, D1, D2, and D3 could dramatically decrease the viability of glioma cells. IC50 of four kinds of derivatives for U87 were 3.99, 3.28, 7.60, and 11.84 μM, respectively. In the U251 cell line, IC50 were 7.00, 5.43, 8.64, and 18.05 μM, respectively (Table 1). D2 and D3, which had better cytotoxicity and lipid-solubility, were chosen for further experiments. EdU assay was used to evaluate the D2 and D3 effects on glioma cell proliferation. As shown in Figures 3C,D, both D2 and D3 could attenuate cell proliferation in a dose-dependent manner.

**TABLE 1:** Brief summary of juglone and derivatives.

Names	Molecular structures	Properties	Molecular weights	IC_50_(μM)	
				U87	U251
Juglone	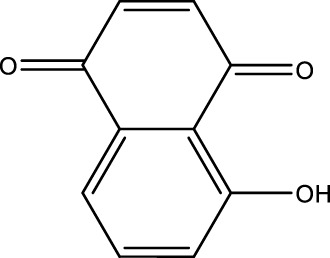	Brown solid	174	27.44	32.04
Juglone-D1	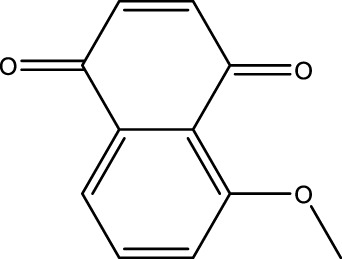	Yellow solid	188	3.99	7.00
Juglone-D2	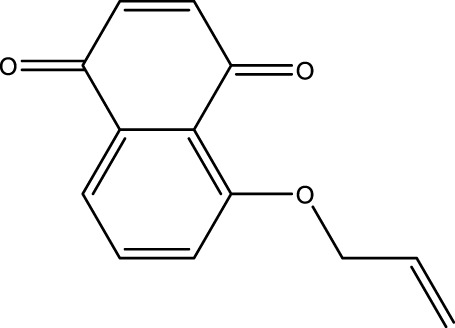	Yellow solid	230	3.28	5.43
Juglone-D3	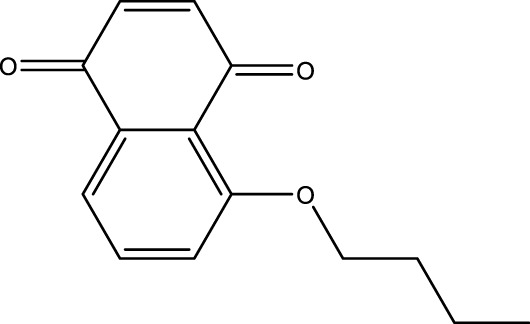	Brown oil	214	7.60	8.64
Juglone-D4	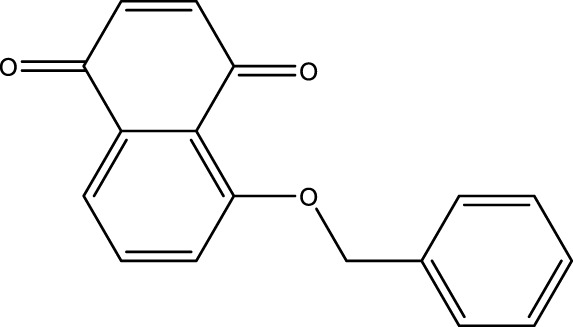	Orange solid	264	11.84	18.05

### Juglone Had a Broad Spectrum of Potential Targets

**FIGURE 4:**
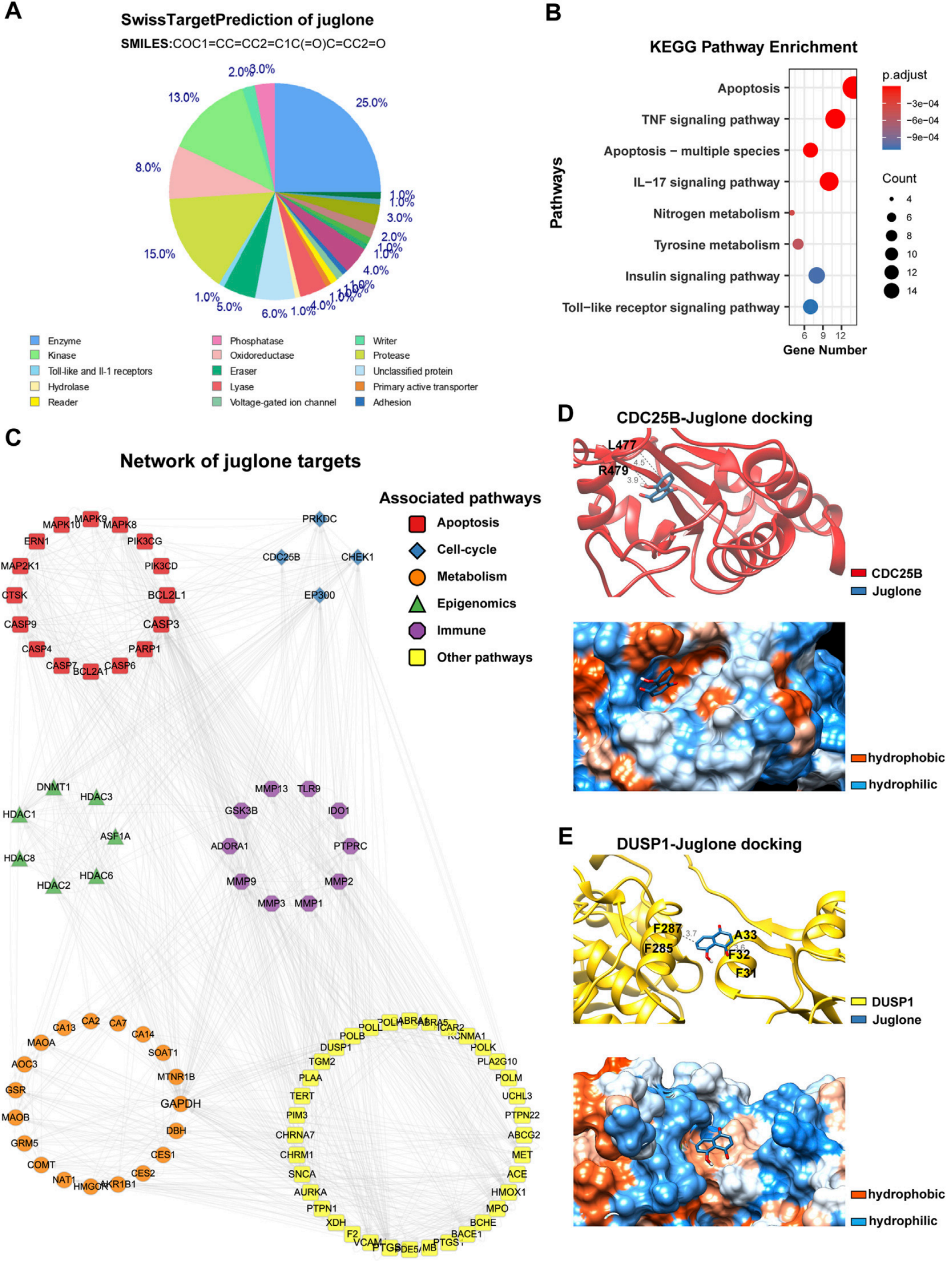
Juglone had a broad spectrum of potential targets. **(A)** The predicted targets of juglone via SwissTargetPrediction. **(B)** KEGG pathway enrichment analysis of the predicted targets of juglone. **(C)** Topological network of juglone targets. Colors of genes are coded for different pathways (apoptosis, cell-cycle, metabolism, epigenomics, immune, and other pathways). **(D)** Molecular docking of juglone with CDC25B. The blue and red ribbons represent the juglone and CDC25B, respectively. The blue and orange dots represent the hydrophilic and hydrophobic amino acids, respectively. **(E)** Molecular docking of juglone with CDC25B. The blue and yellow ribbons represent the juglone and DUSP1, respectively. The blue and orange dots represent the hydrophilic and hydrophobic amino acids, respectively.

To further reveal the underlying mechanism of anti-glioma effects, a pharmaceutical network was established among the predicted targets of juglone. SwissTargetPrediction showed juglone had a broad spectrum of potential targets, most of which are enzymes (25.0%), protease (15.0%), and kinase (13.0%, Figure 4A). KEGG analysis showed apoptosis pathway can be most significantly enriched (Figure 4B). Apart from apoptosis, the protein-protein interaction (PPI) network indicated that juglone could affect biological processes such as cell cycle, metabolism, immune reaction, and epigenomic status to a large extent (Figure 4C). CDC25B and DUSP1 were the two most likely candidates of juglone targets (Table 2), which were reported to be associated with apoptosis (Miyata et al., 2001; Robitaille et al., 2017). Molecular docking further provided interaction details between juglone and these two targets. Figure 4D showed juglone could insert into the pocket of CDC25B and interact with L477 and R479. In the meantime, juglone could protrude into a hydrophobic pocket of DUSP1, and the aromatic ring of juglone could interact with the hydrophobic residues of DUSP1 (A33 and F287) *via* hydrophobicity (Figure 4E).

**TABLE 2:** Juglone targets from SwissTargetPrediction.

Target	Gene symbol	Uniprot id	Target class	Go annotation	Probability
Indoleamine 2,3-dioxygenase	IDO1	P14902	Enzyme	Tryptophan catabolic process to kynurenine	0.739,304
				Regulation of activated T cell proliferation	
Dual specificity phosphatase Cdc25B	CDC25B	P30305	Phosphatase	G2/M transition of mitotic cell cycle	0.739,304
				Protein phosphorylation	
Dual specificity protein phosphatase 1 (by homology)	DUSP1	P28562	Enzyme	Cell cycle	0.221,211
				Cellular response to chemokine	
Histone acetyltransferase p300	EP300	Q09472	Writer	Histone acetylation	0.159,648
				Apoptotic process	
Dual specificity mitogen-activated protein kinase kinase 1	MAP2K1	Q02750	Kinase	MAPK cascade	0.08057
				Cell motility	
Monoamine oxidase B	MAOB	P27338	Oxidoreductase	Dopamine catabolic process	0.08057
Serine/threonine-protein kinase/endoribonuclease IRE1	ERN1	O75460	Enzyme	mRNA cleavage	0.071787
				Protein phosphorylation	
Monoamine oxidase A	MAOA	P21397	Oxidoreductase	Dopamine catabolic process	0.071787
				Cellular biogenic amine metabolic process	
Beta-secretase 1	BACE1	P56817	Protease	Positive regulation of neuron apoptotic process	0.071787
				Amyloid-beta formation	
Hematopoietic cell protein-tyrosine phosphatase 70Z-PEP	PTPN22	Q9Y2R2	Phosphatase	Lipid metabolic process	0.071787
				Autophagy	
Leukocyte common antigen	PTPRC	P08575	Enzyme	Protein dephosphorylation	0.071787
				T cell activation	
Serine/threonine-protein kinase PIM1	PIM1	P11309	Kinase	Apoptotic process	0.071787
				Protein phosphorylation	
Glutathione reductase	GSR	P00390	Oxidoreductase	Cell redox homeostasis	0.071787
				Glutathione metabolic process	

### Juglone Derivatives Could Induce Apoptosis of Gliomas *In Vitro*

**FIGURE 5:**
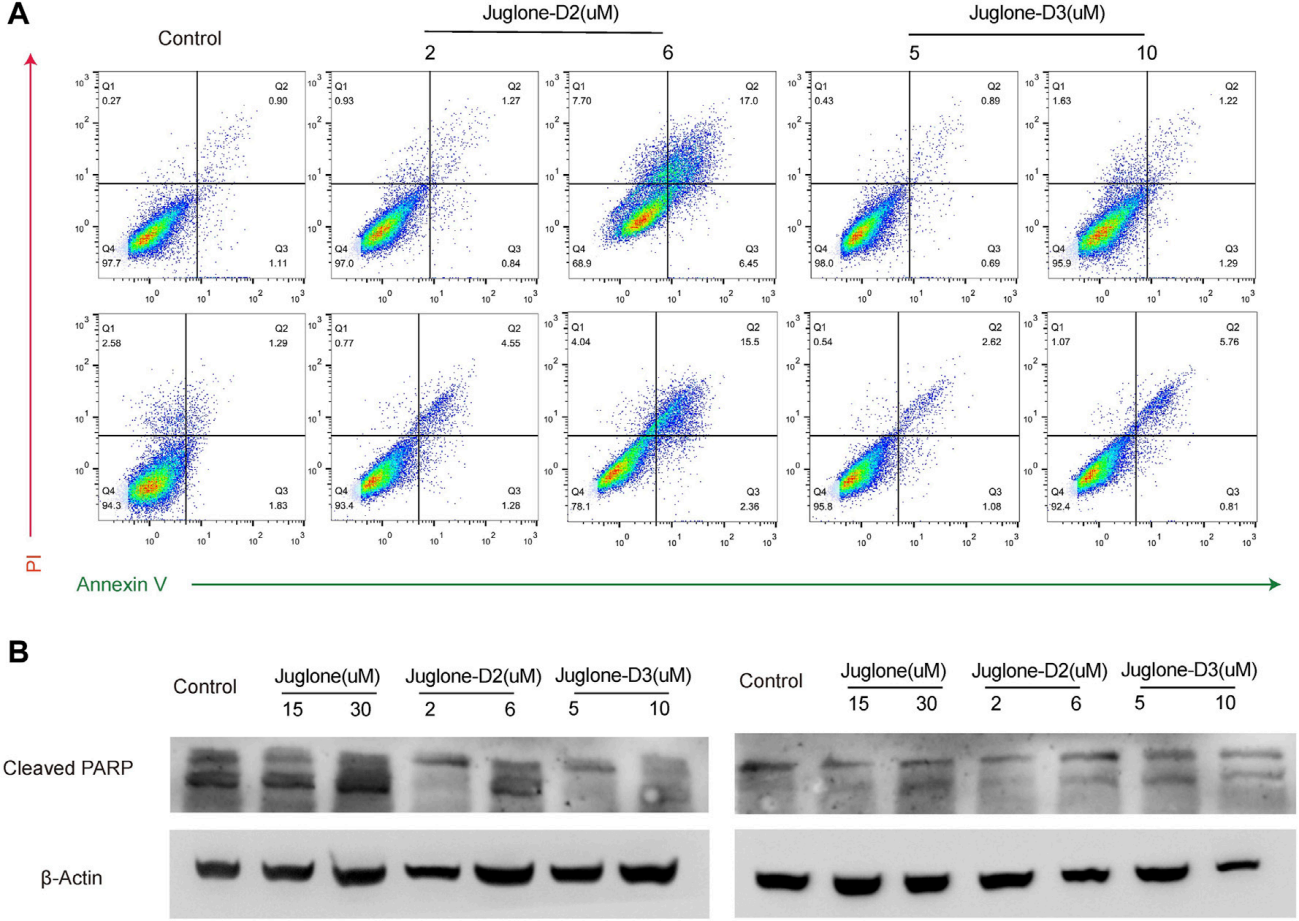
Juglone derivatives could induce apoptosis of glioma cells. **(A)** Flow cytometry detection showed D2 and D3 induced glioma cell apoptosis. **(B)** Western blot assay showed the expression level of cleaved-PARP was increased after D2 or D3 treatment on U87 and U251 cells.

To further validate the effect of juglone derivatives on apoptosis, U87 and U251 cells were stained with Annexin V/PI after treatment of D2 (2 μM, 6 μM) and D3 (5 μM, 10 μM). Flow cytometry analysis showed that D2 and D3 could induce apoptosis and increase the percentage of Annexin V+/PI + cells both in U87 and U251 groups (Figure 5A). To substantiate these phenomena, the expression of cleaved-PARP was evaluated by western blot. As shown, cleaved-PARP was up-regulated after treatment of D2 and D3 in a dose-dependent manner, which indicated these derivatives could induce apoptosis in gliomas (Figure 5B).

### The Cytotoxic Effect Is Dependent on ROS Generation

**FIGURE 6:**
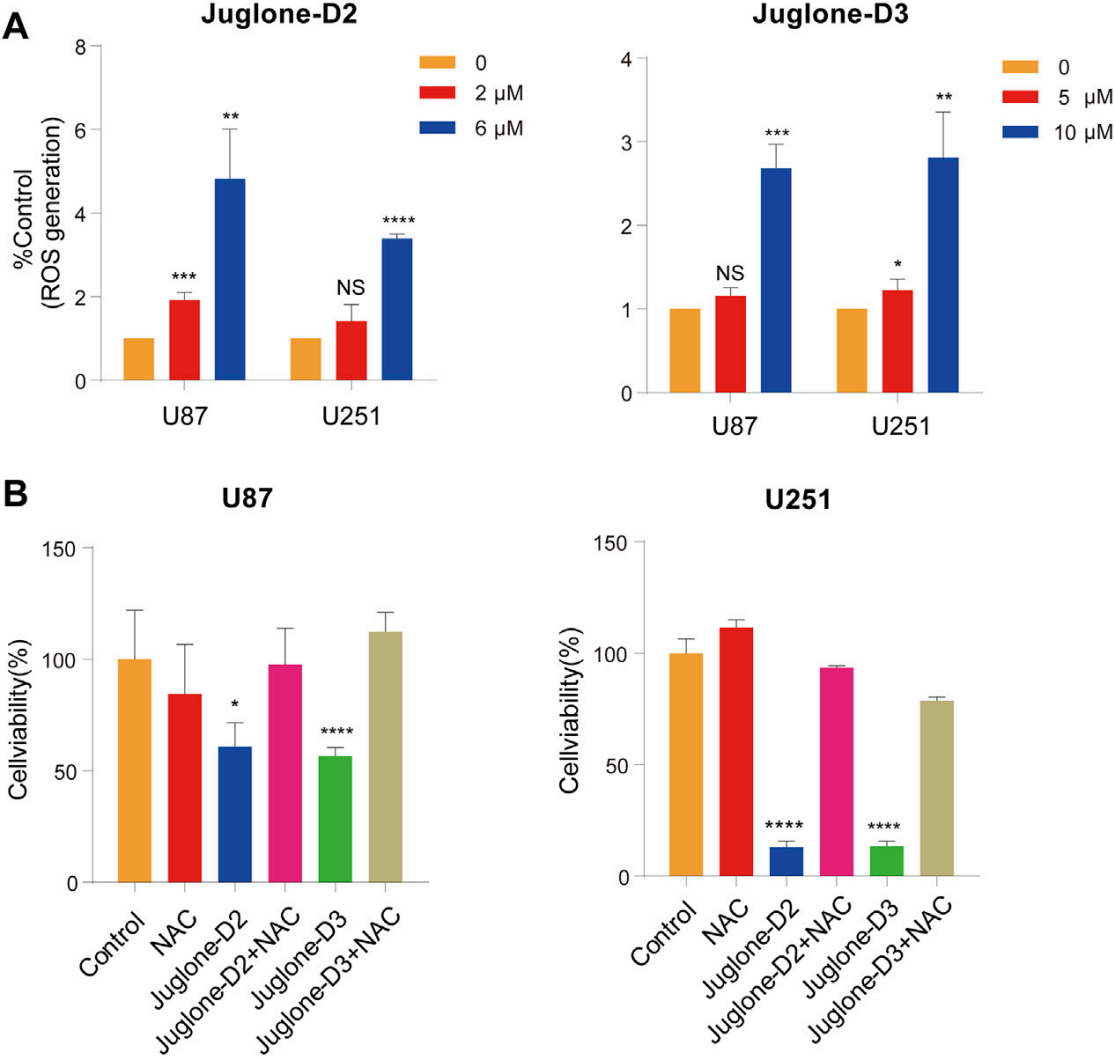
The cytotoxic effect of juglone derivatives is dependent on ROS generation. **(A)** The ROS generation was increased after treatment of D2 and D3. **(B)** NAC, a ROS scavenger, could reverse the cytotoxic effect of ROS. *p<0.05, **p<0.01, ***p<0.001, ****p<0.0001.

As reported in our previous work, juglone could induce ROS generation *via* p38-MAPK pathway activation. In this study, ROS production was also measured with a ROS assay kit by flow cytometry. As demonstrated, D2 and D3 could significantly induce ROS generation in U87 and U251 cells (Figure 6A). In addition, NAC, a ROS scavenger, reversed the cytotoxic effect, indicating the involvement of ROS generation in the anti-glioma effect of D2 and D3 (Figure 6B).

### New Juglone Derivatives Could Exert a Cytotoxic Effect Against Gliomas *In Vivo*

**FIGURE 7:**
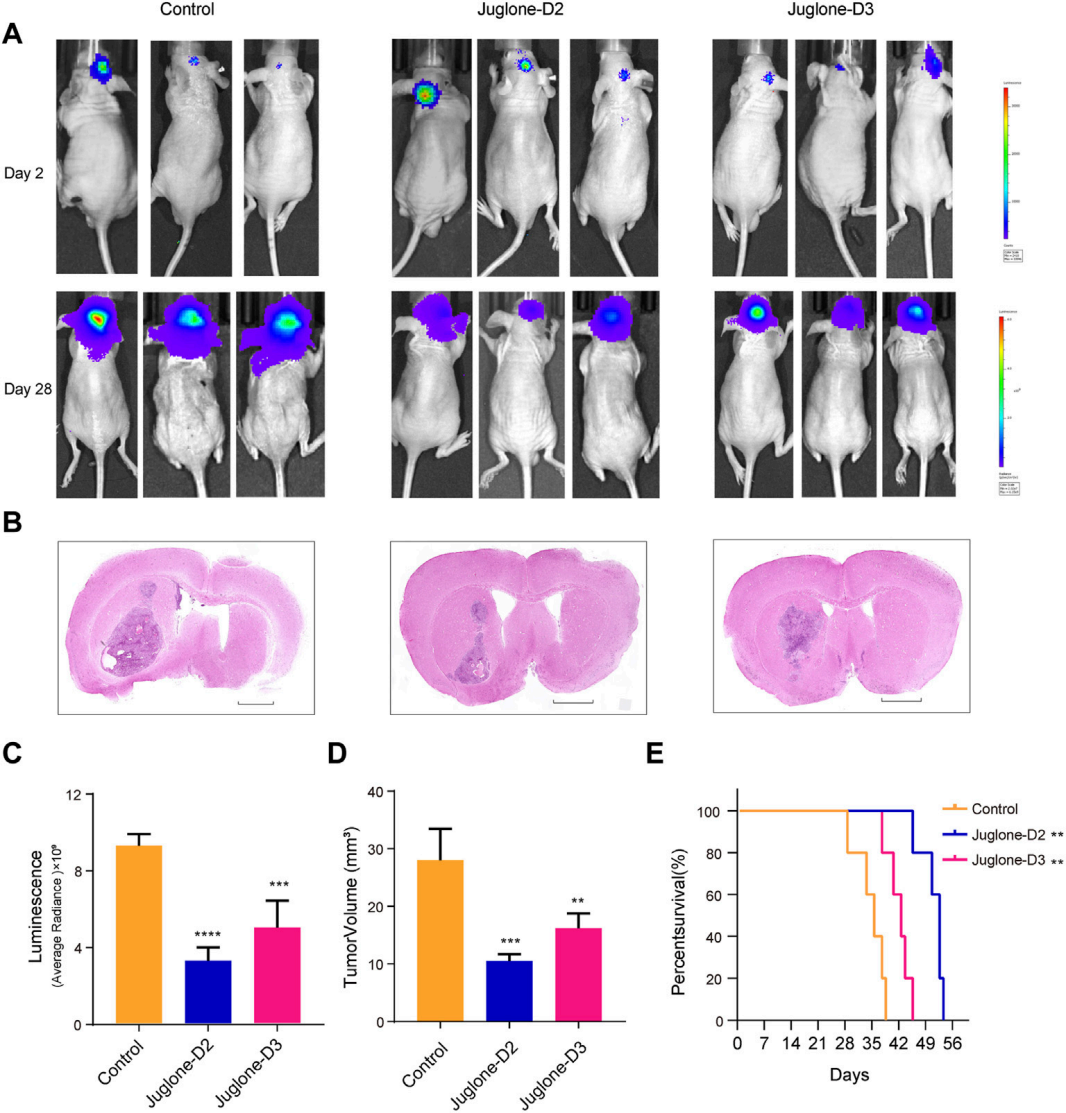
Juglone derivatives could inhibit glioma growth *in vivo*. **(A)** Tumor growth was evaluated by detection of bioluminescence, and revealed D2 and D3 inhibit glioma growth in orthotopic glioblastoma mouse model. **(B)** H and E staining of brain sections confirmed the glioma inhibition effect of D2 and D3. Scale bar = 2 mm. **(C-D)** Luminescence values and tumor volume were measured. **(E)** Kaplan-Meier analysis revealed increased survival of D2 and D3 group relative to controls. **p<0.01, ***p<0.001, ****p<0.0001.

To investigate whether D2 and D3 could effectively inhibit glioma *in vivo*, the orthotopic glioblastoma model was first established (n = 15) and then assigned to the following groups randomly: control (*n* = 5), D2 (*n* = 5), D3 (*n* = 5). Three days after brain implantation of human primary glioma cells infected with lentivirus expressing luciferase into nude mice, vehicle, D2 (1 mg/kg) or D3 (1 mg/kg) were administrated intraperitoneally every other day. Both D2 and D3 had an inhibitory effect on glioma growth confirmed with *in vivo* imaging systems 28 days later after tumor transplantation (Figures 7A,B). HE staining showed that both D2 and D3 could inhibit tumor growth (Figures 7B,D), and no obvious histological harm to the heart and kidney could be observed in the group D2 and D3 compared to the vehicle group. However, there was partial necrosis of liver cells in the D2 and D3 groups (Supplementary Material S5). Kaplan–Meier analysis of survival data demonstrated a statistical difference between the control and the D2 or D3 group (Figure 7E).

## Discussion

Juglone has been widely used in traditional medicine for centuries. Recently, the antitumor property of juglone are reported in many human cancer such as pancreatic cancer (Avci et al., 2016), ovarian cancer (Fang et al., 2015), lung cancer (Zhang et al., 2015), colon cancer (Bayram et al., 2019), and cervical cancer (Lu et al., 2017). Our previous work also demonstrated that juglone could inhibit the proliferation of glioma cells with an IC50 value of 40 μM *via* the reactive oxygen species (ROS) generation mechanism (Wu et al., 2017). Many researchers had studied the anti-glioma effect of juglone before. The EC50 of juglone on rat C6 cells was estimated to be 10.4 ± 1.6 μM (Meskelevicius et al., 2016). Wang et al. investigated the anticancer effect on human U251 cells, and the IC50 in this study was about 50 μM. All these studies showed that only a high concentration of juglone could exert effective cytotoxicity.

It is well-documented that the capability of a substance to penetrate the blood–brain barrier (BBB) into brain parenchyma depends on the biological features and the physicochemical properties of the compound such as molecular weight, hydrogen bonding capacity, and lipophilicity. And the unstable property or the poor BBB penetrating power of juglone hinders its effective use in clinical brain tumor therapy. It is indispensable to block potential oxidation susceptibility to preserve bioactivities. In general, adding halogen or alkyl chemical group could increase the lipophilicity of molecules, facilitating drug active substances crossing the BBB, and entering the central nervous system.

In the current study, we utilize a chemical modification method to substitute the hydroxyl with alkyl, synthesizing different derivatives of juglone, which could increase molecular lipophilicity and better oxidation resistance. We observe the IC50 values of four kinds of derivatives in U87 are 3.99 μM (D1), 3.28 μM (D2), 7.60 μM (D3), and 11.84 μM (D4), compared with 40 μM of juglone in our previous study (Wu et al., 2017). EdU and apoptosis assay reveal new derivatives with allyl (D2) or butyl (D3) substitution of juglone could inhibit proliferation and promote apoptosis of glioma cells effectively *via* the ROS-based pathway. Mice are given an intraperitoneal injection of D2 and D3 at a dose of 1 mg/kg. The dose selection of these two derivatives is based on juglone used in our previous research, as the intraperitoneal median lethal dose is 25 mg/kg according to the manufacturer’s instructions of juglone (Wu et al., 2017). *In vivo* experiment also confirms the anti-glioma effect of D2 and D3 with low cardio-nephrotoxicity. However, hepatotoxicity remains, which needs to be improved in a future experiments. In conclusion, our present study demonstrates that juglone derivatives could exert stronger growth-inhibitory and cytotoxic effects on glioma cells after being modified with an allyl or butyl chemical group substitution.

It is reported that ROS has a dual role in tumor cell progression, as excessive generation of ROS and imbalance of redox reaction results in cell death while moderate increase promotes cell proliferation (Trachootham et al., 2008; Zou et al., 2015). In general, low/physiological concentrations of ROS, like vitamin C, acts as a signal to promote cell survival and prevent DNA injury (Subramani et al., 2014; Velauthapillai et al., 2017). The same phenomenon is also found in our research that juglone at low concentration could promote glioma cell growth, whereas exert anti-glioma effect at high concentration. Hence, ROS-based pathways are well-known mediators in the intracellular signaling cascade, which is also investigated most in the juglone-induced antitumor effect. Marco et al. investigated the voltammetric pattern and confirmed a redox mechanism underlies juglone-induced biological activity in GLI36 human glioma cells (Redaelli et al., 2015). Kastytis etc. revealed that juglone could generate ROS by interacting with mitochondrial respiration in mouse C6 glioma cells (Sidlauskas et al., 2017). It has been proved that excessive levels of ROS production could induce DNA damage, growth arrest, apoptosis, and cell death (Martindale and Holbrook, 2002). Our previous study revealed that juglone could generate a high level of ROS and activate the p38-MAPK pathway, inducing tumor cell apoptosis (Wu et al., 2017). In our current work, we confirm the activation of the p38-MAPK pathway *via* ROS generation is still involved after chemical group modification of juglone. And exogenous antioxidant NAC could diminish the amount of ROS generation of juglone derivatives.

The future drug of these new juglone derivatives surely needs further clinical validation. These novel chemical reagents would be good candidates, especially for those MGMT unmethylated gliomas or recurrent gliomas.

## Data Availability

The data that support the findings of this study are available from the corresponding authors.

## Abbreviations

GBM, glioblastoma; TMZ, temozolomide; TTFields, tumor treating fields; ROS, reactive oxygen species; BBB, blood–brain barrier; NMR, nuclear magnetic resonance; DMEM, Dulbecco’s Modified Eagle Medium; PBS, phosphate buffered saline; RBC, red blood cell; H&E, hematoxylin and eosin; GC-MS, gas chromatography mass spectrometry; and PPI, protein–protein interaction.
