# Supplementary material for: The Anti-Glioma Effect of Juglone Derivatives through ROS Generation
Source: Front Pharmacol. 2022 Jun 14;13:911760. doi: 10.3389/fphar.2022.911760 (PMC9237211; doi:10.3389/fphar.2022.911760)
Supplement: Supplementary file 1 [file Image2.PDF]

Supplementary file 2. Compounds identification and purity test of juglone derivatives.

Juglone-D1

Property: Yellow solid

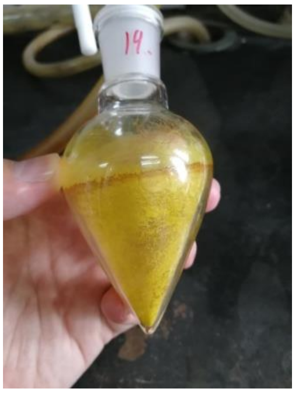

Molecular structure

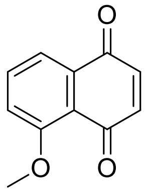

Chemical Formula: $C_{11}H_8O_3$
Exact Mass: 188.04734

MS

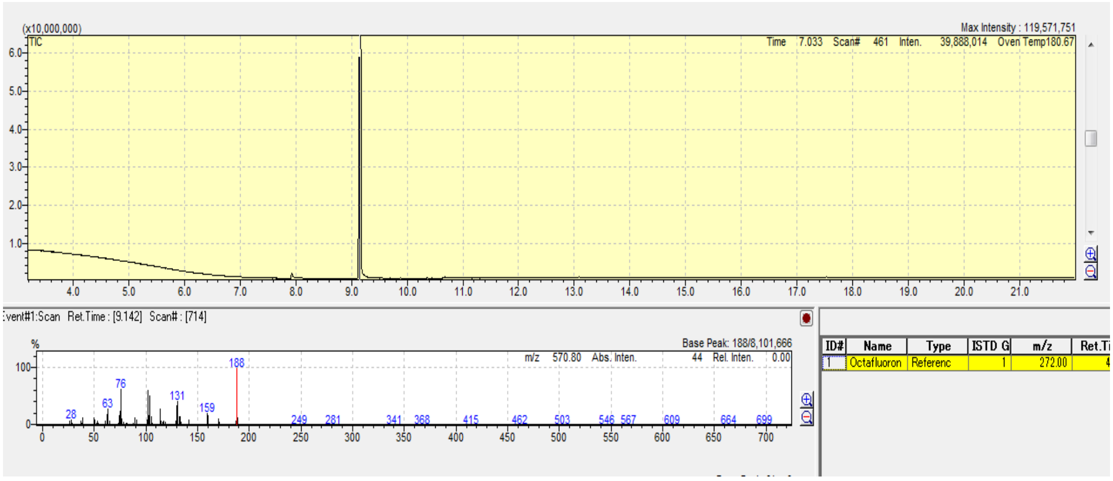

LRMS: m/z calcd for $C_{11}H_8O_3$: 188.04; Found: 188.0.

GC

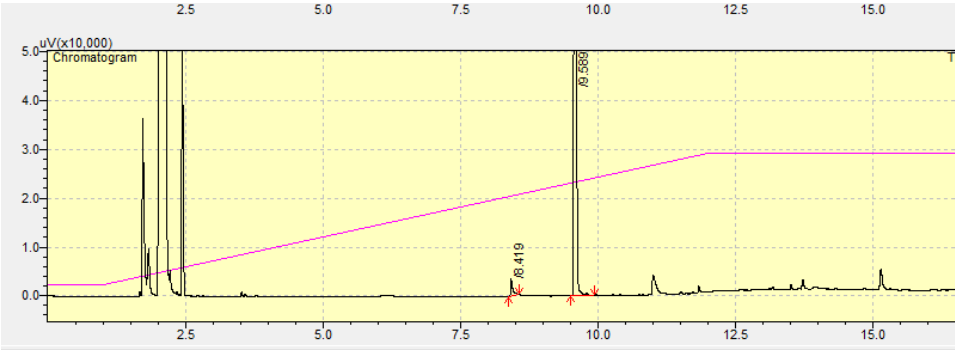

Results - Peak Table

Peak Table | Compound | Group | Calibration Curve

Peak#	Ret.Time	Area	Height	Conc.	Units	Mark	Compound ID#	Compound Name
1	8.419	9731.8	3640.7	0.96159				
2	9.589	1002324.5	512893.3	99.03841				

Supplementary file 2. Compounds identification and purity test of juglone derivatives.

Juglone-D2

Property: Yellow solid

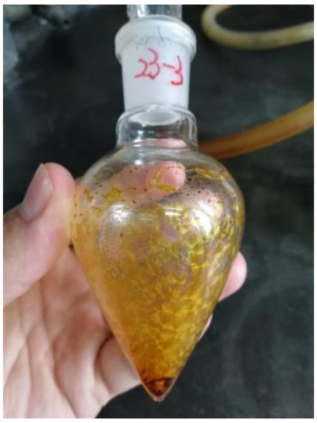

Molecular structure

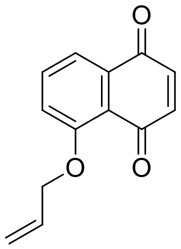

Chemical Formula: $C_{13}H_{10}O_3$
Exact Mass: 214.06299

MS

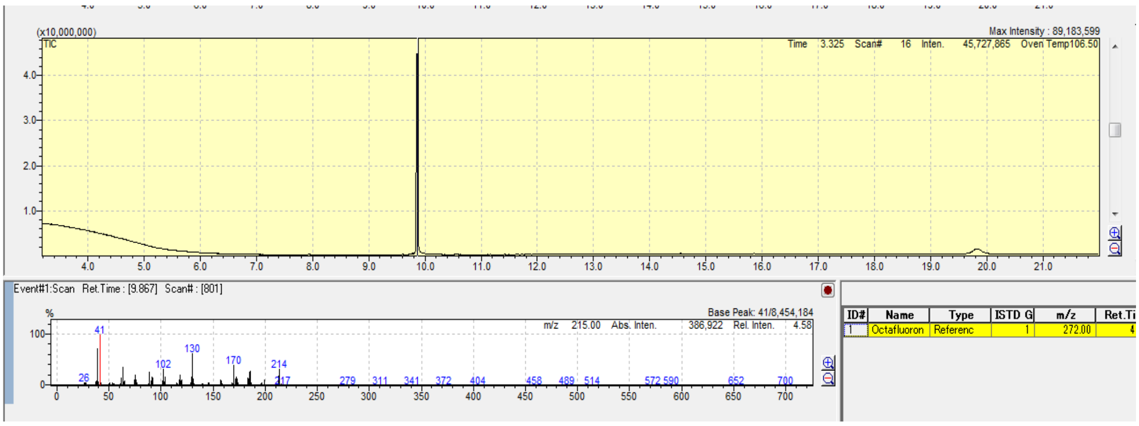

LRMS: m/z calcd for $C_{13}H_{10}O_3$:214.06; Found: 214.0.

GC

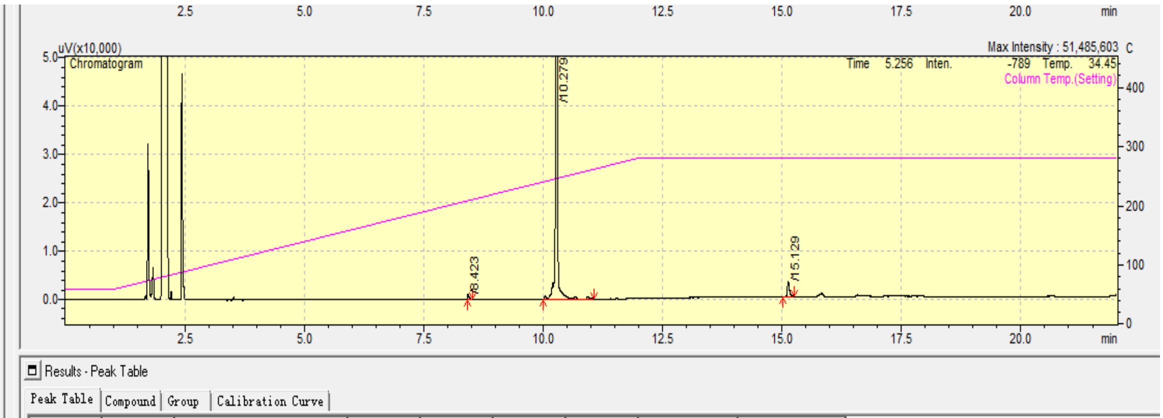

Peak#	Ret.Time	Area	Height	Conc.	Units	Mark	Compound ID#	Compound Name
1	8.423	3034.6	1155.9	0.58554				
2	10.279	505731.2	288295.8	97.58236				
3	15.129	9495.0	3162.6	1.83210				

Supplementary file 2. Compounds identification and purity test of juglone derivatives.

Juglone-D3

Property: Brown oil

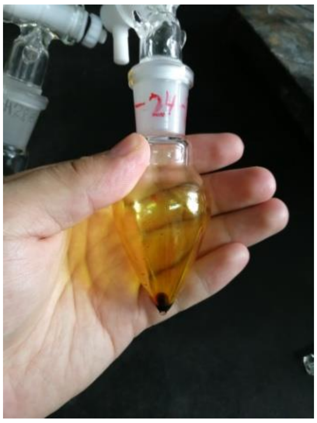

Molecular structure

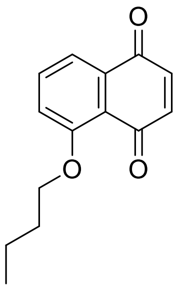

Chemical Formula: $C_{14}H_{14}O_3$
Exact Mass: 230.09429

MS

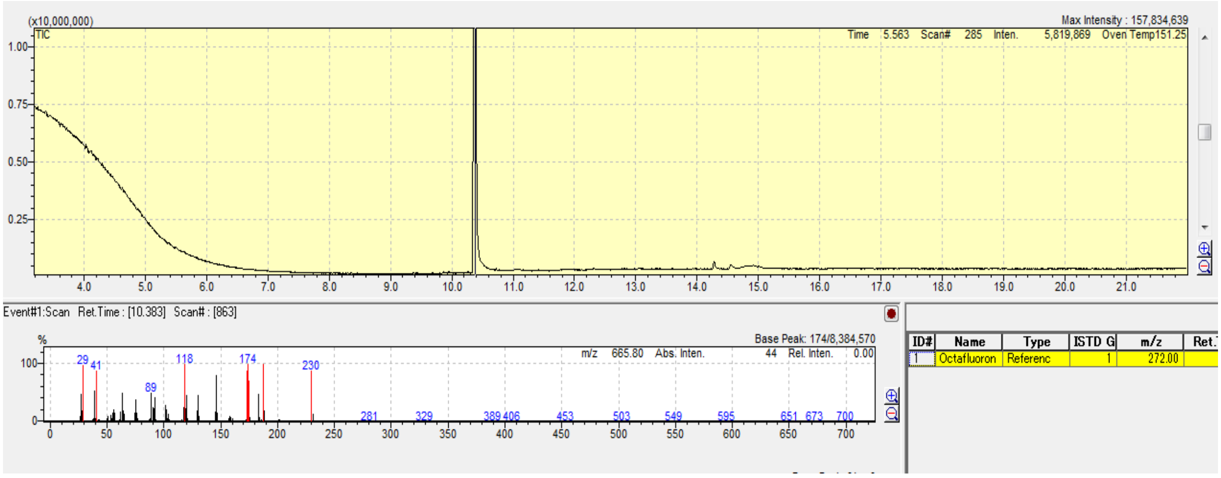

LRMS: m/z calcd for $C_{14}H_{14}O_3$: 230.09 Found:230.0.

GC

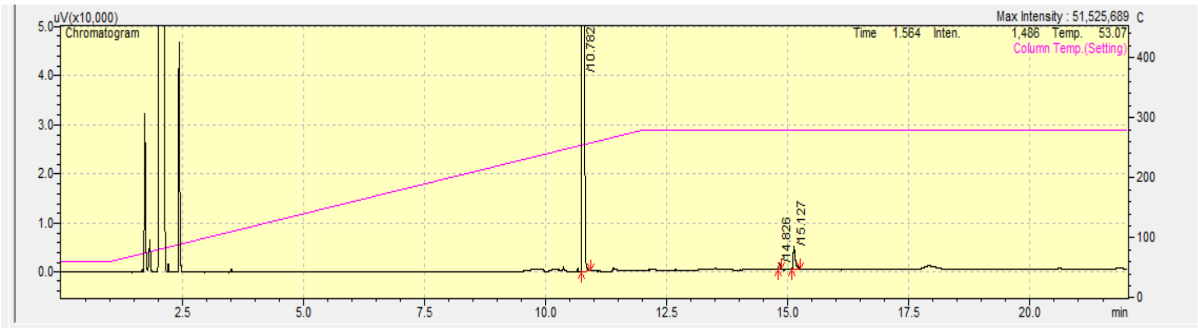

Results - Peak Table

Peak Table | Compound | Group | Calibration Curve

Peak#	Ret.Time	Area	Height	Conc.	Units	Mark	Compound ID#	Compound Name
1	10.782	1117478.1	614906.4	98.52625				
2	14.826	2627.2	1272.6	0.23163				
3	15.127	14088.0	4586.1	1.24212				

Supplementary file 2. Compounds identification and purity test of juglone derivatives.

Juglone-D4

Property: Orange solid

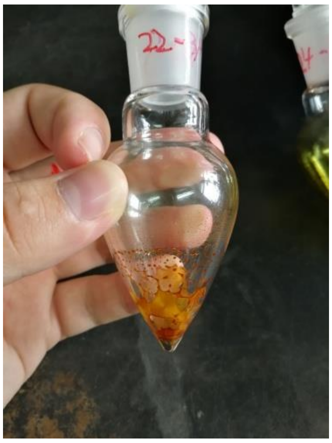

Molecular structure

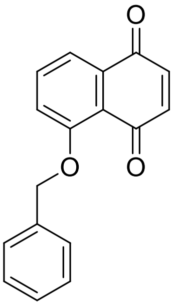

Chemical Formula: $C_{17}H_{12}O_3$
Exact Mass: 264.07864

MS

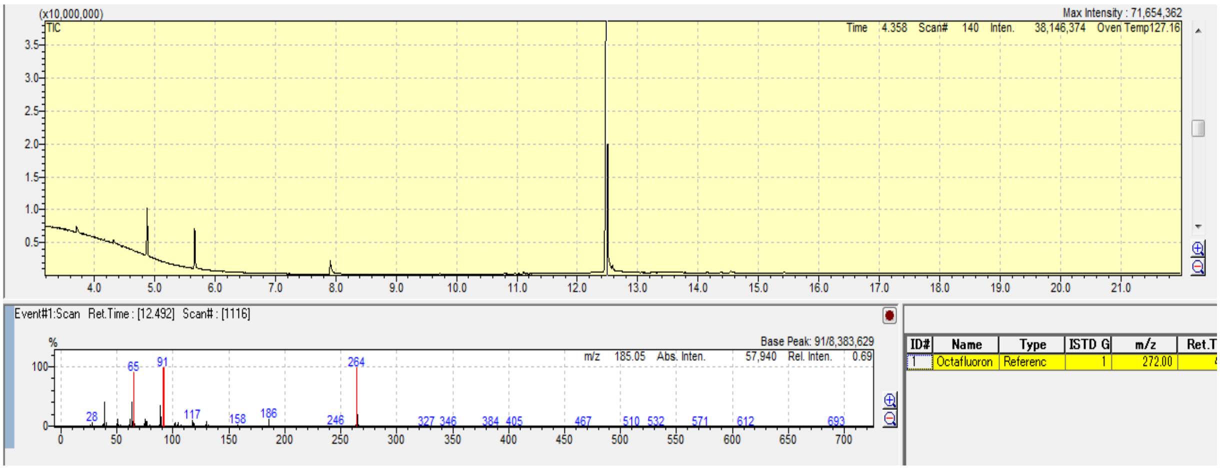

LRMS: m/z calcd for $C_{17}H_{12}O_3$: 264.08; Found: 264.0

GC

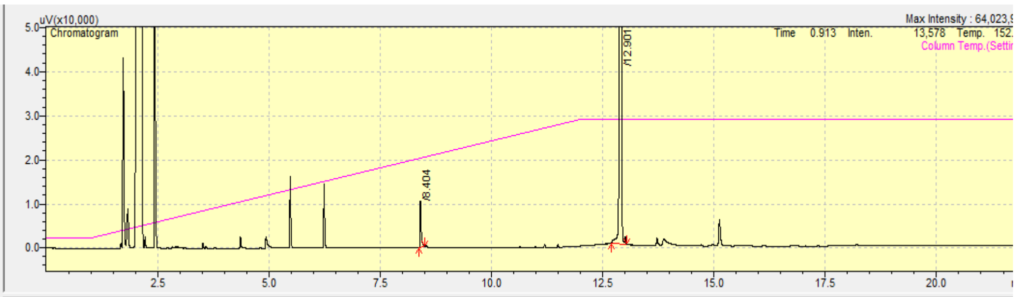

Results - Peak Table

Peak Table | Compound | Group | Calibration Curve

Peak#	Ret.Time	Area	Height	Conc.	Units	Mark	Compound ID#	Compound Name
1	8.404	20235.6	10502.3	2.58010				
2	12.901	764058.7	364919.3	97.41990				
