# Supplementary material for: The Anti-Glioma Effect of Juglone Derivatives through ROS Generation
Source: Front Pharmacol. 2022 Jun 14;13:911760. doi: 10.3389/fphar.2022.911760 (PMC9237211; doi:10.3389/fphar.2022.911760)
Supplement: Supplementary file 2 [file DataSheet1.PDF]

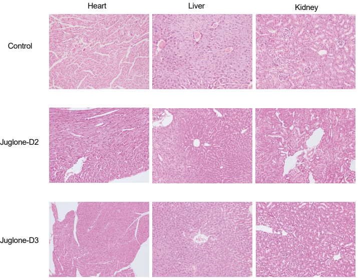

Supplementary 3. H&E staining of heart, liver and kidney. H&E staining revealed that there was slightly necrosis of liver in D2 and D3 group.
