# Supplementary material for: The Anti-Glioma Effect of Juglone Derivatives through ROS Generation
Source: Front Pharmacol. 2022 Jun 14;13:911760. doi: 10.3389/fphar.2022.911760 (PMC9237211; doi:10.3389/fphar.2022.911760)
Supplement: Supplementary file 3 [file Image1.PDF]

Juglone - fresh

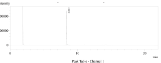

Peak Table - Channel 1

Peak#	Ret.Time	Area	Height	Conc.	Units	Mark	Name
1	8.380	450109	237045	100.000			
Total		450109	237045				

Juglone - 1 day

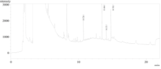

Peak Table - Channel 1

Peak#	Ret.Time	Area	Height	Conc.	Units	Mark	Name
1	10.726	2453	1166	17.009			
2	13.464	5763	2816	39.960			
3	14.121	1121	535	7.774			
4	14.782	5084	1893	35.258			
Total		14421	6410				

Juglone - 1 week

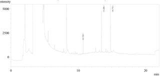

Peak Table - Channel 1

Peak#	Ret.Time	Area	Height	Conc.	Units	Mark	Name
1	10.720	2647	1303	4.888			
2	13.456	8016	4243	14.801			
3	14.772	43494	17756	80.311			
Total		54157	23302				

Supplementary file 1. Gas chromatography-mass spectrometry of juglone in fresh, 1 day and 1 week after dissolution. Juglone was oxidized into different forms.
